# Effect of dexamethasone in patients with ARDS and COVID-19 – prospective, multi-centre, open-label, parallel-group, randomised controlled trial (REMED trial): A structured summary of a study protocol for a randomised controlled trial

**DOI:** 10.1186/s13063-021-05116-9

**Published:** 2021-03-01

**Authors:** Jan Maláska, Jan Stašek, František Duška, Martin Balík, Jan Máca, Jan Hruda, Tomáš Vymazal, Olga Klementová, Jan Zatloukal, Tomáš Gabrhelík, Pavel Novotný, Regina Demlová, Jana Kubátová, Jana Vinklerová, Adam Svobodník, Milan Kratochvíl, Jozef Klučka, Roman Gál, Mervyn Singer, on behalf of the REMED Study Group, Helena Antoni, Petr Suk, Tomáš Korbička, Jan Hudec, Michal Fric, Václav Zvoníček, Tomáš Tencer, Martin Kolář, Petr Kafka, Michal Otáhal, Jan Rulíšek, Marek Flaksa, Eva Svobodová, Peter Sklienka, Filip Burša, Marcela Káňová, Jan Varady, Filip Haiduk, Vladimír Šrámek, Pavel Suk, Marek Fencl, Ivan Čundrle, Pavel Štětka, Marek Lukeš, Miloš Chobola, Jan Beroušek, Zuzana Přikrylová, Jiří Bureš, Jaromír Vajter, Vlasta Vlasáková, Michal Garaj, Lenka Doubravská, Jiří Pouska, Jakub Kletečka, Tomáš Graus, Tereza Šobáňová, Radovan Turek, Tomáš Tyll, Aleš Rára

**Affiliations:** 1 Department of Anaesthesiology and Intensive Care Medicine, University Hospital Brno and Masaryk University, Faculty of Medicine, Jihlavská 20, 625 00 Brno, Czech Republic; 2 Department of Anaesthesia and Intensive Care, University Hospital Královské Vinohrady and Charles University, 3rd Faculty of Medicine, Šrobárova, 1150 100 34 Praha, Czech Republic; 3 Department of Anaesthesia and Intensive Care, 1st Faculty of Medicine, General University Hospital in Prague and Charles University, U Nemocnice 499/2, 128 08 Praha, Czech Republic; 4 Department of Anaesthesiology and Intensive Care Medicine, Faculty of Medicine, University Hospital Ostrava and University Ostrava, 17. listopadu 1790, 708 52 Ostrava-Poruba, Czech Republic; 5 Department of Anaesthesiology and Intensive Care Medicine, Faculty of Medicine, St. Anne’s University Hospital and Masaryk University, Pekařská 664/53, 656 91 Brno, Czech Republic; 6 Department of Anaesthesiology and Intensive Care Medicine, 2nd Faculty of Medicine, University Hospital Motol and Charles University, V Úvalu 84/1, 150 06 Praha 5, Czech Republic; 7 Department of Anaesthesiology and Intensive Care Medicine, Faculty of Medicine, University Hospital Olomouc and Palacky University, I. P. Pavlova 185/6, 779 00 Olomouc, Czech Republic; 8 Department of Anaesthesiology and Intensive Care Medicine, Faculty of Medicine in Pilsen, University Hospital Plzeň and Charles University, alej Svobody 80, 304 60 Plzeň-Lochotín, Czech Republic; 9 Department of Anaesthesiology and Intensive Care Medicine, Tomáš Baťa Regional Hospital, Havlíčkovo nábřeží 600, 762 75 Zlín, Czech Republic; 10 Department of Anaesthesiology and Intensive Care, 1st Faculty of Medicine, Military University Hospital Praha and Charles University, U Vojenské nemocnice 1200, 169 02 Praha, Czech Republic; 11 Department of Pharmacology/CZECRIN, Faculty of Medicine, Masaryk University, Kamenice 5, 62500 Brno, Czech Republic; 12 Department of Paediatric Anaesthesiology and Intensive Care Medicine, Faculty of Medicine, University Hospital Brno and Masaryk University, Jihlavská 20, 625 00 Brno, Czech Republic; 13 Bloomsbury Institute of Intensive Care Medicine, Division of Medicine, University College London, Gower Street, London, WC1E 6BT UK

**Keywords:** COVID-19, Randomised controlled trial, Protocol, ARDS, Dexamethasone, Ventilator-free days

## Abstract

**Objectives:**

The primary objective of this study is to test the hypothesis that administration of dexamethasone 20 mg is superior to a 6 mg dose in adult patients with moderate or severe ARDS due to confirmed COVID-19. The secondary objective is to investigate the efficacy and safety of dexamethasone 20 mg versus dexamethasone 6 mg. The exploratory objective of this study is to assess long-term consequences on mortality and quality of life at 180 and 360 days.

**Trial design:**

REMED is a prospective, phase II, open-label, randomised controlled trial testing superiority of dexamethasone 20 mg vs 6 mg. The trial aims to be pragmatic, i.e. designed to evaluate the effectiveness of the intervention in conditions that are close to real-life routine clinical practice.

**Participants:**

The study is multi-centre and will be conducted in the intensive care units (ICUs) of ten university hospitals in the Czech Republic.

**Inclusion criteria:**

Subjects will be eligible for the trial if they meet all of the following criteria:

1. Adult (≥18 years of age) at time of enrolment;

2. Present COVID-19 (infection confirmed by RT-PCR or antigen testing);

3. Intubation/mechanical ventilation or ongoing high-flow nasal cannula (HFNC) oxygen therapy;

4. Moderate or severe ARDS according to Berlin criteria:

• Moderate – PaO_2_/FiO_2_ 100–200 mmHg;

• Severe – PaO_2_/FiO_2_ < 100 mmHg;

5. Admission to ICU in the last 24 hours.

**Exclusion criteria:**

Subjects will not be eligible for the trial if they meet any of the following criteria:

1. Known allergy/hypersensitivity to dexamethasone or excipients of the investigational medicinal product (e.g. parabens, benzyl alcohol);

2. Fulfilled criteria for ARDS for ≥14 days at enrolment;

3. Pregnancy or breastfeeding;

4. Unwillingness to comply with contraception measurements from enrolment until at least 1 week after the last dose of dexamethasone (sexual abstinence is considered an adequate contraception method);

5. End-of-life decision or patient is expected to die within next 24 hours;

6. Decision not to intubate or ceilings of care in place;

7. Immunosuppression and/or immunosuppressive drugs in medical history:

a) Systemic immunosuppressive drugs or chemotherapy in the past 30 days;

b) Systemic corticosteroid use before hospitalization;

c) Any dose of dexamethasone during the present hospital stay for COVID-19 for ≥5 days before enrolment;

d) Systemic corticosteroids during present hospital stay for conditions other than COVID-19 (e.g. septic shock);

8. Current haematological or generalized solid malignancy;

9. Any contraindication for corticosteroid administration, e.g.

• intractable hyperglycaemia;

• active gastrointestinal bleeding;

• adrenal gland disorders;

• presence of superinfection diagnosed with locally established clinical and laboratory criteria without adequate antimicrobial treatment;

10. Cardiac arrest before ICU admission;

11. Participation in another interventional trial in the last 30 days.

**Intervention and comparator:**

Dexamethasone solution for injection/infusion is the investigational medicinal product as well as the comparator. The trial will assess two doses, 20 mg (investigational) vs 6 mg (comparator). Patients in the intervention group will receive dexamethasone 20 mg intravenously once daily on day 1–5, followed by dexamethasone 10 mg intravenously once daily on day 6–10. Patients in the control group will receive dexamethasone 6 mg day 1–10. All authorized medicinal products containing dexamethasone in the form of solution for i.v. injection/infusion can be used.

**Main outcomes:**

Primary endpoint: Number of ventilator-free days (VFDs) at 28 days after randomisation, defined as being alive and free from mechanical ventilation.

**Secondary endpoints:**

a) Mortality from any cause at 60 days after randomisation;

b) Dynamics of inflammatory marker (C-Reactive Protein, CRP) change from Day 1 to Day 14;

c) WHO Clinical Progression Scale at Day 14;

d) Adverse events related to corticosteroids (new infections, new thrombotic complications) until Day 28 or hospital discharge;

e) Independence at 90 days after randomisation assessed by Barthel Index.

The long-term outcomes of this study are to assess long-term consequences on mortality and quality of life at 180 and 360 days through telephone structured interviews using the Barthel Index.

**Randomisation:**

Randomisation will be carried out within the electronic case report form (eCRF) by the stratified permuted block randomisation method. Allocation sequences will be prepared by a statistician independent of the study team. Allocation to the treatment arm of an individual patient will not be available to the investigators before completion of the whole randomisation process. The following stratification factors will be applied:

• Age <65 and ≥ 65;

• Charlson Comorbidity index (CCI) <3 and ≥3;

• CRP <150 mg/L and ≥150 mg/L

• Trial centre.

Patients will be randomised in a 1 : 1 ratio into one of the two treatment arms. Randomisation through the eCRF will be available 24 hours every day.

**Blinding (masking):**

This is an open-label trial in which the participants and the study staff will be aware of the allocated intervention. Blinded pre-planned statistical analysis will be performed.

**Numbers to be randomised (sample size):**

The sample size is calculated to detect the difference of 3 VFDs at 28 days (primary efficacy endpoint) between the two treatment arms with a two-sided type I error of 0.05 and power of 80%. Based on data from a multi-centre randomised controlled trial in COVID-19 ARDS patients in Brazil and a multi-centre observational study from French and Belgian ICUs regarding moderate to severe ARDS related to COVID-19, investigators assumed a standard deviation of VFD at 28 days as 9. Using these assumptions, a total of 142 patients per treatment arm would be needed. After adjustment for a drop-out rate, 150 per treatment arm (300 patients per study) will be enrolled.

**Trial Status:**

This is protocol version 1.1, 15.01.2021. The trial is due to start on 2 February 2021 and recruitment is expected to be completed by December 2021.

**Trial registration:**

The study protocol was registered on EudraCT No.:2020-005887-70, and on December 11, 2020 on ClinicalTrials.gov (Title: Effect of Two Different Doses of Dexamethasone in Patients With ARDS and COVID-19 (REMED)) Identifier: NCT04663555 with a last update posted on February 1, 2021.

**Full protocol:**

The full protocol (version 1.1) is attached as an additional file, accessible from the Trials website (Additional file 1). In the interest of expediting dissemination of this material, the standard formatting has been eliminated; this Letter serves as a summary of the key elements of the full protocol.

**Supplementary Information:**

The online version contains supplementary material available at 10.1186/s13063-021-05116-9.

## Supplementary Information

**Additional file 1.**

## Data Availability

The sponsor, the trial site and study staff will handle the subject’s personal and trial data according to the effective legislation regarding data protection. Collected data will be shared with other ongoing clinical trials on the same topic for individual patient data (IPD) meta-analysis or shared upon relevant requests. A de-identified participant-level dataset will be made available 6 months after publication of the results of the study at www.mendeley.com

