# Supplementary material for: Effect of dexamethasone in patients with ARDS and COVID-19 – prospective, multi-centre, open-label, parallel-group, randomised controlled trial (REMED trial): A structured summary of a study protocol for a randomised controlled trial
Source: Trials. 2021 Mar 1;22:172. doi: 10.1186/s13063-021-05116-9 (PMC7917377; doi:10.1186/s13063-021-05116-9)
Supplement: Supplementary file 1 — Additional file 1. [file 13063_2021_5116_MOESM1_ESM.pdf]

FAKULTNÍ NEMOCNICE BRNO

CLINICAL TRIAL PROTOCOL

Study Title:

Effect of dexamethasone in patients with ARDS and COVID-19 – prospective, multi-centre, open-label, parallel-group, randomized controlled trial (REMED trial)

**Short title:**	Effect of two different doses of dexamethasone in patients with ARDS and COVID-19
**Protocol code:**	REMED
**EudraCT No.:**	2020-005887-70
**ClinicalTrials.gov Identifier:**	NCT04663555
**Version | Date:**	1.1 | 15.01.2021
**Sponsor:**	Fakultní nemocnice Brno<br>Jihlavská 20<br>625 00 Brno, Czech Republic

This trial protocol contains strictly confidential information and is intended solely for the guidance of the clinical investigation. This protocol may not be disclosed to parties not associated with the clinical research or used for any purpose without the prior written consent of the investigator.

STATEMENT

The following persons undertake to conduct the study **"Effect of dexamethasone in patients with ARDS and COVID-19 – prospective, multi-centre, open-label, parallel-group, randomized controlled trial (REMED trial)"** according to this protocol and in compliance with applicable national and international requirements, mainly:

- Declaration of Helsinki, as amended in 2013;
- Good Clinical Practice according to International Conference on Harmonisation, as amended by ICH E6(R2);
- Act No. 378/2007 Coll., on Pharmaceuticals, as amended;
- Decree No. 226/2008 Coll., on Good clinical practice and detailed conditions of clinical trials on medicinal products, as amended;
- Act No. 18/2018 Coll., on Personal data protection.

		Signature	Date
**Person responsible for the clinical trial on sponsor's side**			
**Investigator responsible for the clinical trial and address of the centre**			

LIST OF TRIAL CENTRES AND PRINCIPLE INVESTIGATORS

Coordinating investigator
Jan Maláska MD, PhD, EDIC
malaska.jan@fnbrno.cz, jan.malaska@gmail.com
+420 532 232 009, +420 532 233 850
Department of Anaesthesiology and Intensive Care Medicine
University Hospital Brno
Jihlavská 20, 625 00 Brno, Czech Republic

Jan Stašek MD, EDIC
stasek.jan@fnbrno.cz, janstasek@centrum.cz
+420 532 232 009
University Hospital Brno
Jihlavská 20
625 00 Brno, Czech Republic

Tomáš Vymazal, MD, PhD, MHA
tomas.vymazal@fnmotol.cz
+420 224 435 401
University Hospital Motol
V Úvalu 84/1
150 06 Praha 5, Czech Republic

František Duška, MD, PhD, AFICM, EDIC
fduska@yahoo.com
+420 267 16 2461
University Hospital Královské Vinohrady
Šrobárova 1150
100 34 Praha, Czech Republic

Olga Klementová, MD, PhD
olga.klementova@fnol.cz
+420 588 443 816
University Hospital Olomouc
I. P. Pavlova 185/6
779 00 Olomouc, Czech Republic

Martin Balík, MD, PhD, EDIC
martin.balik@vfn.cz
+420 224 969 209
General University Hospital in Prague
U Nemocnice 499/2
128 08 Praha, Czech Republic

Jan Zatloukal, MD, PhD
zatloukalj@fnplzen.cz
+420 377 104 386
University Hospital Plzeň
alej Svobody 80
304 60 Plzeň-Lochotín, Czech Republic

Jan Máca, MD, PhD
jan.maca@fno.cz
+420 597 372 713
University Hospital Ostrava
17. listopadu 1790
708 52 Ostrava-Poruba, Czech Republic

Tomáš Gabrhelík, MD, PhD
tomas.gabrhelik@bnzlin.cz
+420 577 552 280
Tomáš Baťa Regional Hospital
Havlíčkovo nábřeží 600
762 75 Zlín, Czech Republic

Jan Hruda, MD, PhD
jan.hruda@fnusa.cz
+420 543 18 3533
St. Anne's University Hospital
Pekařská 664/53
656 91 Brno, Czech Republic

Pavel Novotný, MD, PhD
pavel.novotny@uvn.cz
+420 973 202 999
Military University Hospital Praha
U Vojenské nemocnice 1200
169 02 Praha, Czech Republic

CONTENTS

STATEMENT **2**
LIST OF TRIAL CENTRES AND PRINCIPLE INVESTIGATORS **3**
CONTENTS **4**
LIST OF ABBREVIATIONS **6**
1. CLINICAL TRIAL BACKGROUND *7*
1.1. Introduction 7
1.2. Rationale and up-to-date evidence 7
1.3. Anticipated risks and benefits 7
2. TRIAL OBJECTIVES AND ENDPOINTS *8*
2.1. Primary objective(s) and endpoint(s) 8
2.2. Secondary objective(s) and endpoint(s) 8
2.3. Exploratory objective(s) and endpoint(s) 8
3. TRIAL DESIGN *8*
4. TRIAL POPULATION *9*
4.1. Inclusion criteria 9
4.2. Exclusion criteria 9
4.3. Randomization and stratification 10
4.4. Blinding 10
4.5. Enrolment stopping rules 10
4.6. Premature termination of participation in the trial 10
5. STUDY TREATMENT *11*
5.1. Dexamethasone (ATC code: H02AB02) 11
5.1.1. Qualitative and quantitative composition 11
5.1.2. Pharmaceutical form and route of administration 11
5.1.3. Other information 11
5.2. Packaging and labelling 12
5.3. Delivery and storage 12
5.4. Dosing schedule 12
5.5. Dose modification and delays 12
5.6. Permitted concomitant medication 12
5.7. Restricted concomitant medication 13
6. COURSE OF THE TRIAL *13*
6.1. Screening of eligible patients 13
6.2. Overview of medical care about the study participant 13
6.3. Clinical examinations and assessments 14
6.4. Study procedures 16
6.5. Biological samples 17
6.6. Assessment tools, scores and scales 17
7. SAFETY ASSESSMENTS *17*
7.1. Definitions (according to the Directive 2001/20/EG) 17
7.2. AE surveillance, recording and documentation 18
7.3. Treatment of AE 19
7.4. Assessment of seriousness 19
7.5. Assessment of intensity (severity) 19
7.6. Assessments of causality 19
7.7. Reporting of SAE and SUSAR 20
7.8. Pregnancy 21

8. STATISTICS 21
8.1. Sample size determination 21
8.2. Analysis of efficacy 22
8.2.1. Subgroup analysis 22
8.3. Analysis of safety 22
8.4. Planned interim analysis 22
8.5. Missing data 22
9. DATA MANAGEMENT AND QUALITY ASSURANCE 23
9.1. eCRF database 23
9.2. Trial documents, medical records 23
9.3. Monitoring and auditing 23
9.4. Steering committee 23
9.5. Data monitoring committee 23
10. ETHICAL ASPECTS 24
10.1. Informed consent procedure 24
10.1.1. Fully conscious and oriented patients 24
10.1.2. Patients lacking the capacity to decide 24
10.2. Supervision of the informed consent procedure 25
10.3. Vulnerable population 25
11. PUBLICATION POLICY 25
12. FINANCING AND INSURANCE 25
14. SUPPLEMENTS/APPENDICES 28
14.1. APPENDIX A Specimen of labelling 28
14.2. APPENDIX B Assessment tools, scores and scales 28
14.2.1. APACHE II 28
14.2.2. Barthel index 28
14.2.3. Charlson comorbidity index 28
14.2.4. COV-HI 29
14.2.5. Glasgow coma scale 29
14.2.6. Spontaneous breathing trial 29
14.2.7. WHO Clinical Progression Scale 29

LIST OF ABBREVIATIONS

ADR	Adverse drug reaction
AE	Adverse event
ALT	Alanine aminotransferase
ANOVA	Analysis of variance
ARDS	Acute respiratory distress syndrome
AST	Aspartate aminotransferase
BMI	Body mass index
CCI	Charlson Comorbidity Index
CDK	Chronic kidney disease
COV-HI	COVID-19-associated hyperinflammation
CPS	Clinical Progression Scale
CRF, eCRF	(electronic) Case Report Form
CRP	C-reactive protein
CYP	Cytochrome
ECMO	Extracorporeal membrane oxygenation
EVCTM	EudraVigilance Clinical Trials Module
FiO_2	Inspiratory fraction of oxygen
GCP	Good Clinical Practice
GR	Glucocorticoid receptor
hCG	Human chorionic gonadotropin
HFNC	High Flow Nasal Cannula
ICH	International Conference on Harmonization of Technical Requirements for Registration of Pharmaceuticals for Human Use
ICU	Intensive Care Unit
IPD	Individual patients' data
ITT	Intention to treat
LDH	Lactate dehydrogenase
MedDRA	Medical Dictionary for Regulatory Activities
PEEP	Positive end-expiratory pressure
PP	Per protocol
Ppl	Plateau pressure
RBC	Red blood cells
RR	Respiratory rate
RT-PCR	Polymerase chain reaction with reverse transcription
SADR	Serious adverse reaction
SAE	Serious adverse event
SAP	Statistical Analysis Plan
SARS-CoV-2	Severe Acute Respiratory Syndrome-related Coronavirus 2
SBT	Spontaenous breathing trial
SD	Standard deviation
SmPC	Summary of product characteristics
SpO2	Oxygen saturation
SUSAR	Suspected unexpected serious adverse reaction
UADR	Unexpected adverse reaction
VAP/HAP	Ventilator-associated pneumonia/Hospital-acquired pneumonia
VFD	Ventilator-free day
WBC	White blood cells
WHO	World Health Organization

1. CLINICAL TRIAL BACKGROUND

1.1. Introduction

Since December 2019, SARS-Co-2 virus has infected millions of people worldwide. A significant number of patients develop hyperinflammatory state affecting lungs, which may lead to the need for oxygen therapy. In most severe cases, acute respiratory distress syndrome (ARDS) develops and high flow oxygen therapy or invasive mechanical ventilation is necessary (1). Therapeutic options in coronavirus disease 2019 (COVID-19) associated ARDS patients remain limited and mortality is still excessive.

1.2. Rationale and up-to-date evidence

Systemic corticosteroids have the potential to limit hyperinflammatory response by modulating the immune system. This effect is mediated mainly by binding to the glucocorticoid receptor (GR) α (2). Their effectiveness was proved in heterogeneous ARDS patients recently (3).

In patients with COVID-19 pneumonia in need for oxygen therapy or mechanical ventilation, dexamethasone 6 mg per day is currently recommended. This therapy is mandated by the results of RECOVERY trial (4). After this trial was published, three randomized trials comparing hydrocortisone (5,6) or dexamethasone (7) against placebo were stopped prematurely. All these studies were included in the subsequent individual patients' data (IPD) meta-analysis (8). However, the dose of 6 mg of dexamethasone is currently being reappraised.

The aforementioned study in non-COVID-19 ARDS patients (3) used 20 mg of dexamethasone per day, which is roughly equivalent to the methylprednisolone regimen (1 mg/kg/day) studied in early severe ARDS patients (9). Only these moderate doses (80–100 mg of methylprednisolone, equivalent to 15–19 mg of dexamethasone) have the full potential to modulate the immune response by saturating GRα receptors (2). Importantly, prematurely stopped CoDEX trial (7) comparing dexamethasone against placebo in COVID-19 ARDS patients used an initial daily dose of 20 mg of dexamethasone versus placebo.

In the light of these facts, 6 mg of dexamethasone given to COVID-19 patients with different severity of illness (WHO classification group 5–10) may miss important therapeutic potential or may prevent a potential deleterious effects of a full dose therapeutic corticosteroid. Authors hypothesize that the patients with moderate to severe ARDS undergoing mechanical ventilation may benefit from higher doses of dexamethasone (3,7,9).

1.3. Anticipated risks and benefits

There may or may not benefit from a higher dose of dexamethasone in the improvement of the clinical outcomes of an individual participant. However, there is a potential benefit to society from the participation in this study resulting from insights gained about the dexamethasone use and posology. The potential risks of participating in this study are those associated with dexamethasone use, i.e. known adverse effects of potent glucocorticoids.

2. TRIAL OBJECTIVES AND ENDPOINTS

2.1. Primary objective(s) and endpoint(s)

The **primary objective** of this study is to test the hypothesis that administration of dexamethasone 20 mg is superior over 6 mg in adult patients with moderate or severe ARDS due to confirmed COVID-19.

Primary endpoint: Number of ventilator-free days (VFDs) at 28 days after randomization.

VFDs were defined according to recent recommendations (10):

- Day 0 is the day of randomization
- Time frame is 28 days
- Successful extubation means extubation for > 48 h without reintubation in a 28-d survivor
- Interval reintubations – VFDs are counted from last successful extubation
- If death occurs before Day 28, VFD = 0 (to penalize non-survival, regardless of intubation status)
- If death occurs after Day 28, 28-d ventilation and survival status is used for calculating VFDs (censor after 28 days)

2.2. Secondary objective(s) and endpoint(s)

The **secondary objective** is to investigate the efficacy and safety of dexamethasone 20 mg versus dexamethasone 6 mg.

Secondary endpoints:

a) Mortality from any cause at 60 days after randomization;
b) Dynamics of inflammatory marker (CRP) change from Day 1 to Day 14;
c) WHO Clinical Progression Scale at Day 14 (range 0–10; 0 = no illness, 1–9 = increasing level of care, and 10 = death; APPENDIX B Assessment tools, scores, and scales, Chapter 14.2) (11);
d) Adverse events related to corticosteroids (new infections, new thrombotic complications) until Day 28 or hospital discharge;
e) Independence at 90 days after randomization assessed by Barthel Index (APPENDIX B Assessment tools, scores, and scales, Chapter 14.2).

2.3. Exploratory objective(s) and endpoint(s)

The exploratory objective of this study is to assess **long-term consequences** on **mortality** and **quality of life** at 180 and 360 days.

3. TRIAL DESIGN

REMED is a prospective phase II open-label randomized controlled trial testing superiority of dexamethasone 20 mg vs 6 mg. The study is multi-centre and will be conducted in intensive care units (ICUs) of university hospitals in the Czech Republic. The trial aims to be pragmatic, i.e. designed to evaluate the effectiveness of the intervention in conditions that are very close to the real-life routine clinical practice. Dexamethasone will be administered once daily

intravenously for 10 days. 300 participants will be enrolled and followed up for 360 days after randomization.

4. TRIAL POPULATION

4.1. Inclusion criteria

Subjects will **be eligible** for the trial if they meet all of the following criteria:

1. Adult (≥ 18 years of age) at time of enrolment;
2. Present COVID-19 (infection confirmed by RT-PCR or antigen testing);
3. Intubation/mechanical ventilation or ongoing high-flow nasal cannula (HFNC) oxygen therapy;
4. Moderate or severe ARDS according to Berlin criteria:
    - Moderate – PaO_2/FiO_2 100–200 mmHg;
    - Severe – PaO_2/FiO_2 < 100 mmHg;
5. Admission to ICU in the last 24 hours.

4.2. Exclusion criteria

Subjects will **not be eligible** for the trial if they meet any of the following criteria:

1. Known allergy/hypersensitivity to dexamethasone or excipients of the investigational medicinal product (e.g. parabens, benzyl alcohol);
2. Fulfilled criteria for ARDS for ≥ 14 days at enrolment;
3. Pregnancy or breastfeeding;
4. Unwillingness to comply with contraception measurements from the enrolment to at least 1 week after the last dose of dexamethasone (sexual abstinence is considered as the adequate contraception method);
5. End-of-life decision or patient is expected to die within next 24 hours;
6. Decision not to intubate or ceilings of treatment in place;
7. Immunosuppression and/or immunosuppressive drugs in medical history:
    - **a)** Systemic immunosuppressive drugs or chemotherapy in the past 30 days;
    - **b)** Systemic corticosteroids use before hospitalization;
    - **c)** Any dose of dexamethasone during the present hospital stay for COVID-19 for more than (≥) last 5 days before enrolment;
    - **d)** Systemic corticosteroids during present hospital stay for other conditions than COVID-19 (e.g. septic shock);
8. Present haematological or generalized solid malignancy;
9. Any of contraindications of corticosteroids, e.g.
    - intractable hyperglycaemia;
    - active gastrointestinal bleeding;
    - adrenal gland disorders;
    - a presence of superinfection diagnosed with locally established clinical and laboratory criteria without adequate antimicrobial treatment;
10. Cardiac arrest before ICU admission;
11. Participation in another interventional trial in the last 30 days.

4.3. Randomization and stratification

Randomization will be carried out within the electronic case report form (eCRF) by the stratified permuted block randomization method. The allocation sequences will be prepared by a statistician independent of the study team. Allocation to the treatment arm of an individual patient will not be available to the investigators before the completion of the whole randomization process. Following **stratification factors** will be applied:

- Age < 65 and ≥ 65 (12);
- Charlson Comorbidity index (CCI; APPENDIX B Assessment tools, scores, and scales, Chapter 14.2) < 3 and ≥ 3;
- CRP < 150 mg/L and ≥ 150 mg/L
- Trial centre.

Patients will be randomized in 1 : 1 ratio in one of the two treatment arms. Randomization through eCRF will be available 24 hours every day.

4.4. Blinding

This is an open-label trial, in which the participants and the study staff will learn about the allocated intervention. Blinded pre-planned statistical analysis will be performed (13) according to Chapter 8.

4.5. Enrolment stopping rules

Enrolment of new subjects into the trial will be stopped if any of the following is encountered until the sponsor determines if it is safe for the trial to continue enrolment:

- Death that is related to dexamethasone;
- SAE or SUSAR (see Chapter 7.7) that is related to dexamethasone during 28 days post-administration;
- Emergence of new data that may lead to the trial becoming unethical. This may be merely due to safety concerns of the interventional group (e.g., if harm is reported by other trials).
- After the interim analysis, the Steering committee will review primary outcomes and the summary of adverse events in both arms whilst still blinded to treatment allocation. The enrolment can be stopped if the following criteria are fulfilled:
    a) There is a significant difference in the primary outcome at $p < 0.01$ between the arms.
    b) Futility: The futility criterion is not binding for the Steering committee. Based on available data, the study statistician calculates the probability of being able to reject the null hypothesis with achieving the target number of subjects and the probability of type II error made by stopping the trial prematurely.

4.6. Premature termination of participation in the trial

Reasons for early termination of a patient's participation include:

- Withdrawal of informed consent (subject's decision to withdraw for any reason);
- Important deviation in the process of informed consent (Chapter 10.1);
- Life-threatening adverse reaction to dexamethasone at the discretion of the investigator;

- Newly-emerged pregnancy of a participant after the enrolment;

Subjects **can terminate their participation prematurely** at any time at their request for any reason, but they must notify the investigator. The investigator must contact the sponsor to report the premature discontinuation.

The investigator must:

- Instruct the participant about the right on early termination of participation;
- Assure him/her that the end of participation will not affect the attitude of the physician, or further treatment and its quality;
- Ask for discussing this decision with the investigator in advance;
- Make a note in the patient's medical records and eCRF about the date of early termination of participation.

The **sponsor** reserves the **right to discontinue the study** at any time if there is a significant safety concern (e.g. grade 4 adverse reactions to dexamethasone), or insufficient recruitment despite intensified efforts to enrol patients, or if repeated poor study documentation occurs at a site. The trial can be discontinued by the decision of the **regulatory authority** or **ethics committee**, as well.

5. STUDY TREATMENT

5.1. Dexamethasone (ATC code: H02AB02)

Dexamethasone solution for injection/infusion is the investigational medicinal product as well as the comparator. The trial will assess two doses, 20 mg (investigational) vs 6 mg (comparator).

All authorized medicinal products containing dexamethasone in the form of solution for i.v. injection/infusion can be used.

5.1.1. Qualitative and quantitative composition

Active pharmaceutical ingredient: Dexamethasone sodium phosphate (4 mg/mL)

Excipients: Excipients differ in various dexamethasone solutions for inj./inf. Please see the SmPC of the medicinal product you are using. Several excipients have known biological effects (e.g., allergic reactions): methylparaben, propylparaben, benzyl alcohol. Allergy or hypersensitivity to dexamethasone or excipients is an exclusion criterion (Chapter 4.2).

5.1.2. Pharmaceutical form and route of administration

Solution for injection/infusion; intravenous administration

5.1.3. Other information

Detailed pharmacological profile of the drug, including information on the mechanism of action, contraindications, drug interactions, adverse effects and others can be found in the SmPC.

5.2. Packaging and labelling

The medicinal product will be labelled in accordance with applicable legislation. Specimen of the labelling can be found in APPENDIX A, Chapter 14.1.

5.3. Delivery and storage

The medicinal product containing dexamethasone will be ordered regularly by trial sites' hospital pharmacies and taken from the Czech market. It will be stored up to 30°C in the secondary package to keep it from the light according to the SmPC recommendations.

5.4. Dosing schedule

Patients in the intervention group will receive dexamethasone 20 mg intravenously once daily on day 1–5, followed by dexamethasone 10 mg intravenously once daily on day 6–10.

Patients in the control group will receive dexamethasone 6 mg day 1–10.

5.5. Dose modification and delays

For this study, no dose modification or delays are possible.

Previous treatment with any dose of dexamethasone for more than (≥) 5 days during the present hospital stay for COVID-19 is an exclusion criterion (Chapter 4.2, point 6c). If the study subject is treated with any dose of dexamethasone for less than 5 days before the enrolment, he/she can be enrolled, and there will be no changes in the duration of study drug administration.

5.6. Permitted concomitant medication

Study participants will receive the best **standard of care** according to local protocols and national guidelines, including the state-of-the-art anti-infective and adjunctive treatment of COVID-19, sedation and delirium management, physiotherapy, ventilator, and haemodynamic management.

Currently, the standard of care for hospitalized patients with COVID-19 that require oxygen support (14) can consist of:

- remdesivir (at the discretion of the investigator);
- prevention of thromboembolic disease (anticoagulants);
- gastric ulcer prophylaxis for patients on dexamethasone;
- nutrition, hydration, electrolytes, vitamins, and glucose administration;
- antipyretics (paracetamol, metamizole);
- antibiotics;
- bronchodilators.

All **chronic medication** prescribed to the patient before the enrolment is permitted to continue with the exceptions defined in Chapter 4.2 (point 6) and Chapter 5.7. All **necessary medication** needed during the trial is allowed with exceptions defined in Chapter 5.7.

5.7. Restricted concomitant medication

Systemic corticosteroids other than dexamethasone are restricted, as well as **other investigational medicinal products** currently tested for the treatment of COVID-19 (e.g., investigational antivirals, or monoclonal antibodies). Remdesivir is not considered as an investigational drug, as it has already been authorized.

Concomitant treatment with **potent inhibitors of CYP3A** increases the risk of systemic adverse effects of dexamethasone. In cases where benefits of such treatment do not overweight this increased risk, combination with strong inhibitors of CYP3A is considered restricted.

Potent inhibitors of CYP3A include e.g., systemic azole antimycotics, clarithromycin, erythromycin, telithromycin, diltiazem, and verapamil.

Concomitant use of **potent inducers of CYP3A** decreases the effect of dexamethasone, which may then bias the results. In cases where benefits of such treatment do not overweight this risk of bias, combination with potent inducers of CYP3A is considered restricted.

Potent inducers of CYP3A include e.g., phenytoin, rifampicin, phenobarbital, and carbamazepine.

Detailed information on drug interactions could be found in SmPC.

6. COURSE OF THE TRIAL

6.1. Screening of eligible patients

Patients admitted to ICU of each centre will be screened daily for the eligibility, and all the patients will be recorded in the screening log of eCRF. For patients with the completion of consent, appropriate enrolment information will be recorded to eCRF and only after all required information are provided the randomization and allocation will proceed.

For details on obtaining the informed consent see Chapter 10.1.

6.2. Overview of medical care about the study participant

Patient in both arms will receive the best standard of care. General supportive care guided by official recommendations and local protocols will be given, as described in Chapter 5.6.

The centres are encouraged to follow appropriate antibiotic stewardship practices, e.g. guidelines for hospital-acquired and ventilator-associated pneumonia (VAP/HAP) management (15). As for ventilation strategies, the centres are encouraged to comply with the best evidence of ventilator settings for ARDS patients, i.e. tidal volume approx. 6 mL/kg of predicted body weight, driving pressure (ΔP) < 15 cm of water, plateau pressure (Ppl) < 30 cm of water, setting inspiratory fraction of oxygen (FiO_2) and positive end-expiratory pressure (PEEP) to keep $SaO_2 \geq 90\%$ or $PaO_2 \geq 8$ kPa and set respiratory rate (RR) to maintain pH ≥ 7.20. Beside ventilator settings, other measures are left to the local protocols and at the discretion of each centre, e.g. prone positioning, neuromuscular blocking agents, lung recruitment manoeuvres, and extracorporeal membrane oxygenation (ECMO).

Weaning from mechanical ventilation is performed by the daily screening of readiness through using the spontaneous breathing trial (SBT) described in APPENDIX B Assessment tools, scores, and scales, Chapter 14.2. If SBT is successful and no other significant reason against extubation is present, the patient is extubated. Patients allowed spontaneous ventilation while on ECMO are classified as support dependent until ECMO is weaned off.

6.3. Clinical examinations and assessments

During the trial course, subjects will be monitored during hospitalization and after discharge. A summary of scheduled procedures is outlined in Chapter 6.4. For a graphic summary see Table 1.

Table 1: Clinical trial schedule

	STUDY PERIOD													
	Enrolment	Allocation	Post-allocation						Prim. endpoint	Follow-up				Premature termination
TIMEPOINT	*0–24h*		D_1	D_2	D_3	D_5	D_{10}	D_{14}	D_{28}	D_{60}	D_{90}	D_{180}	D_{360}#	*Anytime*
**ENROLMENT:**														
Inclusion/Exclusion criteria	X													
Informed consent	X													
Pregnancy testing[1]	X													
Randomization		X												
**INTERVENTIONS:**														
Dexamethasone 20 mg			◆	—	—	—	◆							
Dexamethasone 6 mg			◆	—	—	—	◆							
**ASSESSMENTS:**														
Demographic data[2]		X												
Medical history[3]		X												
Vital signs[4]		X	X											
Blood count and routine biochemistry[5]			X											
APACHE II[6]			X											
Ventilatory status			X	X	X	X	X	X	X					X
COV-HI[7]			X	X										
Adverse events			X	X	X	X	X	X	X	X	X	X	X	X
Inflammatory markers[8]			X	X	X	X	X	X						
WHO CPS[9]								X						
Hospitalization status			X	X	X	X	X	X	X					X
Vital status			X	X	X	X	X	X	X	X	X	X	X	X
Barthel index[10]											X	X	X	
Reason for premature termination														X

1 hCG from peripheral blood sample in women of childbearing potential; if statim hCG is not available, perform urine pregnancy testing
2 Age, sex, race or ethnic group, BMI
3 Allergies, comorbidities, and Charles comorbidity index (CCI, Chapter 14.2.3), chronic medication
4 Temperature, blood pressure, heart rate, respiratory rate
5 Blood count (RBC, WBC, haematocrit, lymphocyte count, platelets), coagulation profile, renal and liver functions (BUN, creatinine, bilirubin, AST, ALT), D-dimer, LDH, troponin, pH, blood gases, sodium, potassium, chloride, glycaemia (other laboratory tests and imaging methods are left at the discretion of each centre)
6 Counted using the worst values in the first 24 h of ICU stay – for details See APPENDIX B, Chapter 14.2.1
7 D_1 – CRP, ferritin, D_2 – CRP; COV-HI is defined as CRP ≥ 150 mg/L or doubling within 24 h from greater than 50 mg/L, or ferritin ≥ 1500 µg/L (12,16,17)
8 CRP (other markers e.g. procalcitonine, presepsin, interleukin-6 at the discretion of each centre)
9,10 See APPENDIX B, Chapter 14.2

\# End of trial visit (or phone call)

6.4. Study procedures

At enrolment

- Eligibility screen – inclusion and exclusion criteria
- Informed consent procedure (Chapter 10.1)
- Pregnancy testing (hCG) in women with childbearing potential – preferentially from blood sample, if statim hCG is not available at the time of enrolment, urine pregnancy testing is necessary (see Chapter 7.8)
- Demographic data (age, sex, race or ethnic group, BMI)
- Medical history (allergies, comorbidities and CCI, chronic medication)
- Vital signs (temperature, blood pressure, heart rate, respiratory rate)

Day 1

- Vital signs (temperature, blood pressure, heart rate, respiratory rate)
- Administration of dexamethasone according to the allocation to treatment arms
- Blood count and routine biochemistry [RBC, WBC, haematocrit, lymphocyte count, platelets, coagulation profile, renal and liver functions (BUN, creatinine, AST, ALT, bilirubin), D-dimer, LDH, troponin, pH, blood gases, sodium, potassium, chloride, glycaemia]
- Inflammatory markers (CRP; other markers e.g., procalcitonine, presepsin, interleukin-6 at the discretion of each centre)
- COV-HI assessment (APPENDIX B, Chapter 14.2.4) – based on Day 1 CRP or ferritin level;
- APACHE II score counted using the worst values in the first 24 h of ICU stay (the score is determined based on specific parameters from medical history, vital signs, blood count and biochemistry, and other examinations; APPENDIX B, Chapter 14.2.1)

Day 2

- Administration of dexamethasone according to the allocation to treatment arms
- Inflammatory markers (CRP; other markers e.g., procalcitonine, presepsin, interleukin-6 at the discretion of each centre)
- COV-HI assessment (APPENDIX B, Chapter 14.2.4) – based on Day 2 CRP level.

Day 3 to Day 10

- Administration of dexamethasone according to the allocation to treatment arms
- Inflammatory markers (CRP; other markers e.g., procalcitonine, presepsin, interleukin-6 at the discretion of each centre)

Day 14

- World Health Organization Clinical Progression Scale (APPENDIX B, Chapter 14.2.7)
- Inflammatory markers (CRP; other markers e.g., procalcitonine, presepsin, interleukin-6 at the discretion of each centre)

Day 1 to Day 28 / ICU discharge (whichever comes first)

- Checking adverse events and adverse reactions (with special interest in newly emerged infections and new thrombotic complications)
- Ventilatory status, i.e. use of a mechanical ventilator or other ventilation/oxygen support

- Vital status
- Discharge from ICU and hospital

Days 60, 90, 180, 360

- Out-patient visits (if possible) OR structured phone call interviews lead by study nurse with the patient or his/her family member or caregiver
- Vital status
- Quality of life by the assessment of daily life activities and functional independence (Barthel Index; APPENDIX B, Chapter 14.2.2)
- Checking adverse events and reactions

6.5. Biological samples

Blood samples will be taken following local practice and analysed in the respective clinical laboratory facility of the trial centre. No other biological specimens will be obtained or stored in this trial.

6.6. Assessment tools, scores, and scales

The following tools will be used during the trial and are described in APPENDIX B Assessment tools, scores, and scales, Chapter 14.2):

- APACHE II
- Barthel index
- Charlson comorbidity index
- COV-HI
- Glasgow coma scale
- Spontaneous breathing trial
- WHO CPS

7. SAFETY ASSESSMENTS

Before the start of the clinical trial, all Investigators will undergo training in pharmacovigilance requirements and obtain detailed written instructions for recording and reporting of AEs. These instructions for pharmacovigilance will be part of the Investigator's Site File.

7.1. Definitions (according to the Directive 2001/20/EG)

For clinical trials, effective legislation has introduced the following definitions.

Table 2: Definitions of safety signals in clinical trials

<table>
<tr><td>Adverse event</td><td>AE</td><td>Any untoward medical occurrence in a patient or clinical trial subject administered a medicinal product and which does not necessarily have a causal relationship with this treatment.</td></tr>
<tr><td>Adverse drug reaction</td><td>ADR</td><td>All untoward and unintended responses to an investigational medicinal product related to any dose administered</td></tr>
<tr><td>Serious adverse event</td><td>SAE</td><td rowspan="2">A serious adverse event/reaction is any untoward medical occurrence or effect that at any dose:
• Results in death;
• Is life-threatening;
• Requires hospitalization or extension of existing hospitalization;
• Results in persistent or significant disability or incapacity;
• Is a congenital anomaly or birth defect.</td></tr>
<tr><td>Serious adverse reaction</td><td>SADR</td></tr>
<tr><td>Unexpected adverse reaction</td><td>UADR</td><td>Adverse reaction, the nature, severity, or outcome of which is not consistent with the product information (SmPC)</td></tr>
<tr><td>Suspected unexpected serious adverse reaction</td><td>SUSAR</td><td>Any suspected adverse reaction related to the study treatment that is both serious and unexpected.</td></tr>
</table>

7.2. AE surveillance, recording and documentation

AE could be diseases or symptoms which occur or worsen after the enrolment of a patient in the clinical trial. All AEs need to be documented, no matter if the investigator suspects a causal connection to the study medication. AE will be monitored and documented **from the day of giving informed consent until the end of participation in the study** (i.e., Day 360).

Subjects will be instructed to report any AEs that they experience to the investigator. The investigator should actively ask about AEs.

Each AE should be described, documented in the eCRF, and evaluated to determine:

- Seriousness (see Chapter 7.4);
- Severity (see Chapter 7.5);
- Causality, i.e. relation to the study medication (see Chapter 7.6.);
- Duration (start and end dates or whether it continues).
- Action taken (no action taken; study medication discontinued; prolongation of the ongoing hospitalization; administration of a drug etc.)

AE needs to be **followed until its resolution**, i.e. until it subsides, stabilizes, becomes chronic, or the subject dies.

If AE fulfils the criteria of SAE, a separate form must be completed besides the standard eCRF record (see Chapter 7.7).

AEs will be recorded according to the Medical Dictionary for Regulatory Activities (MedDRA). The most recent MedDRA version at the start of the study will be used.

7.3. Treatment of AE

A patient with an AE must receive appropriate therapy. The investigator may decide to discontinue the study medication. The patient will remain under medical supervision until the investigator believes that the AE has been resolved.

7.4. Assessment of seriousness

AE is considered serious if it fulfils the definition in Chapter 7.1.

Some situations can be **considered as SAE even if they do not fulfil the criteria** of the definition. These are important medical events that may not be immediately life-threatening, or result in death, or hospitalization but may jeopardize the subject, or may require intervention to prevent one of the other outcomes listed in the definition above. These should also be considered SAE – e.g., allergic bronchospasm, convulsions, or other states requiring treatment.

If AE fulfils the criteria of SAE, a separate **Serious Adverse Event Form** must be completed and sent to the sponsor. For the procedure see Chapter 7.7.

In this clinical trial, the following SAE are excluded from the notification requirement:

- SAE which occurs after enrolment (i.e., after giving informed consent), but before the study medication was initiated;
- Hospitalization(s) planned before the enrolment;
- Death not related to the dexamethasone use.

7.5. Assessment of intensity (severity)

The intensity (severity) of an AE should be evaluated according to these 5 categories:

**Grade 1**	AE is asymptomatic or mildly symptomatic, requires only observation, no medical intervention
**Grade 2**	AE with medium intensity, requires local, non-invasive, or small-scale treatment
**Grade 3**	AE is medically significant, requires hospitalization or extension of ongoing hospitalization, but it is not directly life-threatening
**Grade 4**	AE is life-threatening and requires urgent significant medical intervention
**Grade 5**	AE leads to death

7.6. Assessments of causality

To assess the relation between administration of the study medication and the AE, the following definitions apply:

- **Related –** The event is known to occur with the study medication, there is a reasonable possibility that the study medication caused the AE, or there is a temporal relationship between the study medication and AE. Reasonable possibility means that there is evidence to suggest a causal relationship between the study medication and the AE.

- **Not related** – There is not a reasonable possibility that the administration of the study medication caused the AE, there is no temporal relationship between the study medication and AE onset, or an alternate aetiology has been established.

7.7. Reporting of SAE and SUSAR

The Investigator is obliged to report any SAE within 24 hours after he/she learns about it to the Sponsor using Serious Adverse Event Form (SAE Form). The blank forms are stored in the Investigator's Site File. The announcement will be done by e-mail: farmakovigilance@med.muni.cz AND nerusilova@med.muni.cz.

If at that point all required information is not available, succeeding records will be sent by the investigator. Sponsor will check the notification completeness and formal plausibility. If required, queries will be made and followed up.

In the case of death, a copy of the autopsy record should be added. If the death of the subject complies with the definition of SUSAR (see Chapter 7.1), it will be reported as SUSAR (see below).

Sponsor has full responsibility for the safety of the clinical trial. Further reporting of AEs to the competent authorities according to the legal requirements is the responsibility of the sponsor.

Follow-up

All SAEs should be followed until their resolution, i.e., they subside, stabilize, become chronic, or the subject dies followed. In case of early termination because of SAE occurrence, the subject should be followed until SAE resolution. All necessary extra visits will be recorded in the eCRF as "Unscheduled visits".

Follow-up information is sent using a new SAE Form stating that this is a follow-up to the previously reported SAE and giving the date of the original report. Within 24 hours of receipt of follow-up information, the investigator must update the SAE form and submit any supporting documentation (e.g., laboratory test reports, subject discharge summary, autopsy report etc.) The follow-up information should describe whether the event has resolved or continues, if and how it was treated and whether the patient continued or withdrew from trial participation.

Reporting of SUSAR

SAE related to the study medication fulfilling the criterion of unexpectedness (i.e., SUSAR) must be reported **by the sponsor** to the Ethics Committee and the EudraVigilance database (module EVCTM), at the latest 15 days after it becomes known. Sponsor will also inform all Investigators involved in the trial. Reporting to the EudraVigilance database will be performed *via* regulatory authority according to the agreement between the sponsor and the regulatory authority. In case of a fatal or life-threatening SUSAR, the sponsor will report all relevant

information immediately, at the latest 7 days after the event becomes known. Any subsequent additional information is forwarded within the next 8 days if necessary.

7.8. Pregnancy

Pregnant or breastfeeding women cannot be included in the study. Pregnancy testing (hCG, blood sample) is obligatory at enrolment in women of childbearing potential (i.e. from menarche to the onset of postmenopausal state). In case the information regarding menstrual cycle cannot be obtained from the patient due to her health or consciousness status, pregnancy testing is also necessary independently of patient's age. If statim hCG is not available at the moment of enrolment, investigators should perform urine pregnancy testing. Urine test strips will be supplied to the trial centres, where statim hCG is not available, e.g. on weekends or public holidays.

Participants, men and women, must comply with the required contraception measurements from the enrolment to at least 1 week after the last dose of dexamethasone. Sexual abstinence is considered as the adequate contraception method for this clinical trial. Newly-emerged pregnancy in the hospitalization phase of the trial is highly unlikely. However, if it is diagnosed, dexamethasone treatment must be stopped. If a participant becomes pregnant during the follow-up phase, she must inform the investigator.

The Investigator should report the pregnancy to the Sponsor within 24 hours of learning of its occurrence using Pregnancy Form. This announcement will be done by e-mail: farmakovigilance@med.muni.cz AND nerusilova@med.muni.cz.

Pregnancy should be followed by the investigator until completion. If it ends for any reason before the anticipated date, the investigator should notify the sponsor. At the completion of the pregnancy, the investigator will document the outcome of the pregnancy. If the outcome of the pregnancy meets the criteria for classification as SAE (e.g., spontaneous abortion, stillbirth, neonatal death, postpartum complication, or congenital anomaly), the investigator should follow the procedures for reporting an SAE (see Chapter 7.7).

8. STATISTICS

A separate Statistical Analysis Plan (SAP) and Statistical Interim Analysis Plan (SIAP) will be prepared to provide details on the approach to analyses. The SAP will be finalized before the interim database lock. All eventual deviations from the SAP will be described and justified in the relevant part of The Clinical Trial Report.

8.1. Sample size determination

The sample size was calculated to detect the difference of 3 VFDs at 28 days (primary efficacy endpoint) between the two treatment arms at two-sided type I error of 0.05 and power of 80%. Based on data from a multi-centre randomized controlled trial in COVID-19 ARDS patients in Brazil (7) and the multi-centre observational study from French and Belgian ICUs regarding moderate to severe ARDS related to COVID-19 (18), investigators assumed standard deviation of VFD at 28 days as SD=9. Using these assumptions, a total of 142 patients per

treatment arm would be needed, after adjustment for drop-out rate, 150 per treatment arm (300 patients per study) will be enrolled.

8.2. Analysis of efficacy

Primary efficacy endpoint (number of VFDs at day 28) will be calculated for both treatment arms separately with corresponding 95% confidence intervals. Comparison between arms will be based on parametric or non-parametric test (following the type of data distribution) and will be adjusted for relevant baseline covariates (stratification parameters)

Secondary and exploratory efficacy endpoints will be analysed following the type of data (Chi-square test or Fisher's exact test for binary data and ANOVA or a non-parametric alternative tests for continuous and ordinal data, if appropriate).

The primary population for analysis of efficacy will be Intention-To-Treat population (ITT), and results will be confirmed on the Per-Protocol Population (PP).

8.2.1. Subgroup analysis

Pre-planned subgroup analysis will be performed regarding the primary outcome variable in the subgroups defined by the following criteria:

- Age
- Sex
- BMI
- Comorbidities
- P/F ratio
- Length of dexamethasone treatment before enrolment
- ECMO procedure

No adjustments of p-values due to multiplicity are planned.

8.3. Analysis of safety

The safety will be analysed in all patients who received any dose of study treatment and provided at least one post-dose safety assessment (Safety Population). All adverse events will be coded and tabulated by system organ class and preferred term for individual events within each system organ class and will be presented in descending frequency. Adverse events will also be tabulated by severity and relationship to the study medication. Serious adverse events will be summarized separately.

8.4. Planned interim analysis

One interim analysis is planned for safety and efficacy evaluation after the primary outcome is known for the 150th subject (50% of planned sample size). To control the overall level of type I error, the P value of 0.01 for interim analysis and 0.04 for the final analysis will be applied. All the details on stopping rules for interim analysis will be described in SIAP.

The interim analysis will be evaluated and decision about continuation of the study will be done by DMC.

8.5. Missing data

No imputation techniques for missing data will be applied.

9. DATA MANAGEMENT AND QUALITY ASSURANCE

9.1. eCRF database

All participants will be assigned an identification code to ensure the pseudonymization of their data. The investigator will maintain a subject identification list for the trial centre (subject identification codes with the corresponding subject names) to enable records to be identified.

Trial data will be collected in eCRFs managed in electronic data capture system REDCap. Access to the database (username, password) will be granted by the study data manager, and the respective study staff will be trained for using it right and safely. The investigator is responsible for the data correctness, completeness, and filling in time. eCRF will be designed to generate queries on missing data or unusual data entries, which will be fed back to the study site investigators in regular intervals.

9.2. Trial documents, medical records

The sponsor, the trial site and study staff will handle the subject's personal and trial data according to the effective legislation regarding data protection. Any paper or electronic trial documents or data are confidential and must not be disclosed to the third persons. In the informed consent form, the participants are informed that their medical records can be provided only to the authorized monitors, auditors, or inspectors.

Medical records of the subjects will be retained in the trial site for 15 years (from the end of the clinical trial), so will be the relevant trial administrative documents at the sponsor's side.

9.3. Monitoring and auditing

The trial centre will be monitored according to the Monitoring plan. The objectives of the monitoring are to ensure that the trial participant's safety and rights are respected, that accurate, valid and complete data are collected, and that the trial is conducted in accordance with the trial protocol, the principles of GCP and national legislation. The investigator agrees that the monitor will regularly visit the trial centre and will be given appropriate support (e.g., the access to all necessary documents incl. patient's medical records). A report on the progress, findings and resolution of any discrepancies will be prepared from each monitoring visit. The investigator undertakes to read the monitoring report and to ensure that any possible discrepancies are corrected. Sponsor, Regulatory authority and Ethics committees have the right to inspect/audit the trial site. The investigator undertakes to co-operate will the inspectors/auditors.

9.4. Steering committee

The Steering Committee is constituted by all study investigators of the REMED trial. It is responsible for the development of the study protocol, continuous and final results interpretations and manuscripts preparation. The Steering Committee closely collaborates with independent Data monitoring committee and the sponsor.

9.5. Data monitoring committee

Members of data monitoring committee (DMC) are experts in intensive care medicine which are not involved in the REMED trial and an independent statistician. DMC is responsible for performing interim analysis and for providing recommendations to the Steering Committee and

the sponsor regarding the safety and continuation of the trial based on evidence of possible significant differences between intervention and control group. Members of DMC are listed in a separate document.

10. ETHICAL ASPECTS

This trial will be conducted following the applicable legislation and requirements for good clinical practice according to the ICH E6(R2). Compliance with this standard provides public assurance that the rights, safety and well-being of trial participants are protected and that the clinical trial data are credible. All essential trial documents and their potential amendments will be submitted to the relevant Ethics committee and Regulatory authority for approval.

10.1. Informed consent procedure

The investigator assesses patient’s ability to decide and extent of potential consciousness impairment based on GCS and other appropriate clinical measures (at discretion of the trial centre).

10.1.1. Fully conscious and oriented patients (GCS 15)

Patient with decision-making capacity will go through the standard procedure (informative interview with the investigator, written information for the patients, the possibility to ask questions, and adequate time to discuss with family and decide). If the patient wishes to participate, he/she will provide written prospective informed consent.

10.1.2. Patients with limited ability to decide (GCS 14 or 13)

Some patients may be limited in their decisional capacity due to their acute health status, or medication. Generally, if a patient understands simplified information and can communicate verbally, the simplified procedure of obtaining informed consent will be applied. The shortened (one-page) information sheet and consent form for signature will be used.

As soon as the patient regains full decisional capacity, he/she will be approached to provide consent with the continuation of his/her participation in the trial. Patients will be informed about the option to withdraw from the trial. Patients who decide to terminate their involvement can permit the sponsor to use the data collected, or they can ask for deleting all data collected. Both options will be presented to them.

If the patient does not regain a decisional capacity, the initial consent will remain valid.

10.1.3. Patients lacking the capacity to decide (GCS 12 or less)

It is expected that a significant proportion of screened patients will lack the capacity to provide informed consent due to severely altered consciousness, severe respiratory distress, or sedation necessary to facilitate mechanical ventilation. In this situation, the deferred consent policy will be applied. Such a patient will be enrolled after independent physician witnesses (in writing) that the patient cannot give his/her consent and fulfils eligibility criteria.

Patient's close person (spouse/partner, close relative, caregiver) will be informed about the patient's enrolment and the nature of the study. If possible and compliant with the epidemiological restrictions by the government, patient's close person will meet the investigator

for an informative interview, to obtain the information leaflet, and to sign a confirmation that he/she was informed about the patient's participation in the trial.

As soon as the patient regains decisional capacity, he/she will be approached to provide consent with the continuation of his/her participation in the trial. Patients will be informed about the option to withdraw from the trial. Patients who decide to terminate their involvement can permit the sponsor to use the data collected, or they can ask for deleting all data collected. Both options will be presented to them.

If the patient does not regain a decisional capacity, the initial consent by an independent physician will remain valid.

10.2. Supervision of the informed consent procedure

The process of obtaining informed consent from a patient or an independent physician must always be appropriately documented by the investigator through valid forms and the patient's medical records, as well. The clinical trial monitor will check the process during the monitoring visits. Important deviations in the process will lead to the termination of the patient’s participation in the trial.

10.3. Vulnerable population

Beside patients with diminished decision capacity, other specifically vulnerable participants (children, pregnant women, prisoners, refugees, institutionalized patients, patients with severe mental illnesses, etc.) will not be enrolled in this clinical trial.

11. PUBLICATION POLICY

Results of this clinical trial are planned to be published in the medical literature. Any publications must be approved by the sponsor and meet the quality requirements for current clinical research publications (e.g., CONSORT statement).

12. FINANCING AND INSURANCE

REMED is an investigator-initiated clinical trial. Funding will be granted from the project research infrastructure Czech Clinical Research Infrastructure Network CZECRIN (LM 2018128) and University Hospital Brno. Trial funders have no role in the study design, collection, analysis and interpretation of data. Investigators declare no financial or non-financial competing interest regarding the focus of this trial.

Mandatory insurance of the participants is arranged. The coverage for damages emerging from the participation in the clinical trial will be provided according to the applicable legal requirements.

13. REFERENCES

1. Wu C, Chen X, Cai Y, Xia J, Zhou X, Xu S, et al. Risk Factors Associated With Acute Respiratory Distress Syndrome and Death in Patients With Coronavirus Disease 2019 Pneumonia in Wuhan, China. JAMA Intern Med. 2020 01;180(7):934–43.

2. Meduri GU, Annane D, Confalonieri M, Chrousos GP, Rochwerg B, Busby A, et al. Pharmacological principles guiding prolonged glucocorticoid treatment in ARDS. Intensive Care Med. 2020 Dec;46(12):2284–96.

3. Villar J, Ferrando C, Martínez D, Ambrós A, Muñoz T, Soler JA, et al. Dexamethasone treatment for the acute respiratory distress syndrome: a multicentre, randomised controlled trial. Lancet Respir Med. 2020;8(3):267–76.

4. RECOVERY Collaborative Group, Horby P, Lim WS, Emberson JR, Mafham M, Bell JL, et al. Dexamethasone in Hospitalized Patients with Covid-19 - Preliminary Report. N Engl J Med. 2020 Jul 17;

5. Angus DC, Derde L, Al-Beidh F, Annane D, Arabi Y, Beane A, et al. Effect of Hydrocortisone on Mortality and Organ Support in Patients With Severe COVID-19: The REMAP-CAP COVID-19 Corticosteroid Domain Randomized Clinical Trial. JAMA. 2020 06;324(13):1317–29.

6. Dequin P-F, Heming N, Meziani F, Plantefève G, Voiriot G, Badié J, et al. Effect of Hydrocortisone on 21-Day Mortality or Respiratory Support Among Critically Ill Patients With COVID-19: A Randomized Clinical Trial. JAMA. 2020 06;324(13):1298–306.

7. Tomazini BM, Maia IS, Cavalcanti AB, Berwanger O, Rosa RG, Veiga VC, et al. Effect of Dexamethasone on Days Alive and Ventilator-Free in Patients With Moderate or Severe Acute Respiratory Distress Syndrome and COVID-19: The CoDEX Randomized Clinical Trial. JAMA. 2020 06;324(13):1307–16.

8. WHO Rapid Evidence Appraisal for COVID-19 Therapies (REACT) Working Group, Sterne JAC, Murthy S, Diaz JV, Slutsky AS, Villar J, et al. Association Between Administration of Systemic Corticosteroids and Mortality Among Critically Ill Patients With COVID-19: A Meta-analysis. JAMA. 2020 06;324(13):1330–41.

9. Meduri GU, Golden E, Freire AX, Taylor E, Zaman M, Carson SJ, et al. Methylprednisolone infusion in early severe ARDS: results of a randomized controlled trial. Chest. 2007 Apr;131(4):954–63.

10. Yehya N, Harhay MO, Curley MAQ, Schoenfeld DA, Reeder RW. Reappraisal of Ventilator-Free Days in Critical Care Research. Am J Respir Crit Care Med. 2019 01;200(7):828–36.

11. WHO Working Group on the Clinical Characterisation and Management of COVID-19 infection. A minimal common outcome measure set for COVID-19 clinical research. Lancet Infect Dis. 2020;20(8):e192–7.

12. Barda N, Riesel D, Akriv A, Levy J, Finkel U, Yona G, et al. Developing a COVID-19 mortality risk prediction model when individual-level data are not available. Nat Commun. 2020 07;11(1):4439.

13. Harhay MO, Casey JD, Clement M, Collins SP, Gayat É, Gong MN, et al. Contemporary strategies to improve clinical trial design for critical care research: insights from the First Critical Care Clinical Trialists Workshop. Intensive Care Med. 2020;46(5):930–42.

14. Therapeutic Management [Internet]. COVID-19 Treatment Guidelines. [cited 2020 Dec 14]. Available from: https://www.covid19treatmentguidelines.nih.gov/therapeutic-management/

15. Torres A, Niederman MS, Chastre J, Ewig S, Fernandez-Vandellos P, Hanberger H, et al. International ERS/ESICM/ESCMID/ALAT guidelines for the management of hospital-acquired pneumonia and ventilator-associated pneumonia: Guidelines for the management of hospital-acquired pneumonia (HAP)/ventilator-associated pneumonia (VAP) of the European Respiratory Society (ERS), European Society of Intensive Care Medicine (ESICM), European Society of Clinical Microbiology and Infectious Diseases (ESCMID) and Asociación Latinoamericana del Tórax (ALAT). Eur Respir J. 2017;50(3).

16. Keddie S, Ziff O, Chou MKL, Taylor RL, Heslegrave A, Garr E, et al. Laboratory biomarkers associated with COVID-19 severity and management. Clin Immunol Orlando Fla. 2020;221:108614.

17. Manson JJ, Crooks C, Naja M, Ledlie A, Goulden B, Liddle T, et al. COVID-19-associated hyperinflammation and escalation of patient care: a retrospective longitudinal cohort study. Lancet Rheumatol. 2020 Oct;2(10):e594–602.

18. Grimaldi D, Aissaoui N, Blonz G, Carbutti G, Courcelle R, Gaudry S, et al. Characteristics and outcomes of acute respiratory distress syndrome related to COVID-19 in Belgian and French intensive care units according to antiviral strategies: the COVADIS multi-centre observational study. Ann Intensive Care. 2020 Oct 6;10(1):131.

14. SUPPLEMENTS/APPENDICES

14.1. APPENDIX A Specimen of labelling

EudraCT No. 2020-005887-70 Klinické hodnocení **REMED**
Zadavatel: Fakultní nemocnice Brno, Jihlavská 20, 625 00 Brno

DEXAMETHASON (ATC KÓD: H02AB02)
Koncentrace: 4 mg/ml **| Léková forma:** inj/inf sol

Pouze pro účely klinického hodnocení.
Pokyny pro užívání a uchování přípravku – viz SPC a Protokol

Kód pacienta: **Léčebné rameno:** 20 | 06

Centrum KH: název, adresa

Zkoušející: jméno | e-mail | telefon

14.2. APPENDIX B Assessment tools, scores, and scales

14.2.1. APACHE II

The APACHE-II score provides an estimate of ICU mortality based on several laboratory values and patient signs taking both acute and chronic disease into account. The score consists of the following parameters: age, severe organ insufficiency/immunodeficiency, body temperature, mean arterial pressure, heart rate, RR, oxygenation, arterial pH, serum Na, K, creatinine, haematocrit, WBC, Glasgow coma scale. More information could be found here: https://www.mdcalc.com/apache-ii-score

Reference: Knaus W. A., Draper E. A., Wagner D. P., Zimmerman J. E. APACHE II: a severity of disease classification system. *Crit Care Med* 1985; 13 (10): 818–829.

14.2.2. Barthel index

The Barthel Index evaluates functional limitations in 10 activities of daily living: food intake, dressing, locomotion, stair walk, moving from bed to chair, personal hygiene, bathing, toilet use, and urine and stool continence. The assessment is performed by a healthcare professional. For details please see: https://www.uzis.cz/index.php?pg=registry-sber-dat--klasifikace--barthelove-test

Reference: Mahoney F. I., Barthel D. Functional evaluation: the Barthel Index. *Maryland State Med Journal* 1965; 14: 56–61.

14.2.3. Charlson comorbidity index

The index quantifies an individual's burden of disease and corresponding mortality risk. It is counted from information on age, cardiovascular, neurological, respiratory, gastrointestinal, metabolic, kidney and oncological diseases, or AIDS. More information could be found here: https://www.mdcalc.com/charlson-comorbidity-index-cci

Reference: Charlson M. E., Pompei P., Ales K. L., MacKenzie C.R. A new method of classifying prognostic comorbidity in longitudinal studies: development and validation. *J Chronic Dis* 1987; 40: 373–383.

14.2.4. COV-HI

A part of COVID-19 patients have a high-risk hyperinflammatory phenotype. The diagnostic criteria for COVID-19-associated hyperinflammation are CRP ≥ 150 mg/L or doubling within 24 h from greater than 50 mg/L or a ferritin ≥ 1500 µg/L. COV-HI is associated with adverse outcomes, e.g. mortality and need for escalation of respiratory support.

Reference: Manson J. J., Crooks C., Naja M., Ledlie A., Goulden B., Liddle T., et al. COVID-19-associated hyperinflammation and escalation of patient care: a retrospective longitudinal cohort study. *Lancet Rheumatol* 2020;2 (10): e594–602.

14.2.5. Glasgow coma scale

The Glasgow coma scale describes the level of consciousness of patients. It consists of the assessment of eye opening, verbal manifestation and motor activity of the patient. For details please see: https://www.glasgowcomascale.org/

Reference: Teasdale G., Jennett B. Assessment of coma and impaired consciousness. A practical scale. *Lancet* 1974; 2: 81–84.

14.2.6. Spontaneous breathing trial

Spontaneous breathing trial tests the ability of a patient to breathe while recieving minimal or no ventilatory support. The accepted modes are disconnection of patient's artificial airway from the ventilator while providing adequate humidity and temperature of inhaled air or keeping the patient on ventilator with minimal level of Positive End-Expiratory Pressure (PEEP of 5 cm H_2O) and Pressure Support (PS of 5 cm H_2O)

Reference: Yang K, Tobin MJ, A prospective study of indexes predicting the outcome of weaning from mechanical ventilation. *N Engl J Med* 1991; 324: 1445–1450

14.2.7. WHO Clinical Progression Scale

The scale provides a measure of illness severity across a range from 0 (not infected) to 10 (dead). It assesses the clinical state of a patient regarding vital status, confirmation of virus RNA, symptoms of infection, hospitalization and the way of breathing support.

Reference: WHO Working Group on the Clinical Characterisation and Management of COVID-19 infection. A minimal common outcome measure set for COVID-19 clinical research. *Lancet Infect Dis* 2020; 20 (8): e192–7.
